# Hospital Meetings

**Published:** 1904-12-24

**Authors:** 


### HOSPITAL MEETINGS.

### CITY OF LONDON TRUSS SOCIETY.

A special general meeting of the City of London Truss Society was held on Wednesday, December 14th, Mr. W. H. Laurie presiding, when certain alterations were made in the rules relating to the privileges of subscribers and donors. Hitherto, it was explained, donors of 10 guineas or upwards at one time had been entitled to recommend four patients annually, while donors of five guineas had recommended two patients. By substituting the word subscribers for donors, and limiting the number of letters to two and one respectively, it was proposed that these privileges should in future be curtailed, that the same restriction should apply to public companies and partnership firms; that executors should be governors for 15 years instead of for life; and that clergymen and ministers should be entitled to recommend one patient instead of two, in cases where the lending of the pulpit resulted in a collection of five guineas. The alterations having been approved by the meeting, the opinion was expressed that in order still further to tend towards economy, the number of letters to which annual subscribers of one guinea were entitled should be also curtailed, since on Mr. John Langton's showing every subscriber of one guinea received letters to the value of 31s. or 32s. A special resolution was passed to the effect that the alterations in the rules should not come into operation before January 1905, and should take effect on donations, life subscriptions, legacies and ch urch collections received on and after that date only.

